# Explaining presenteeism behaviour with the theory of planned behaviour – a longitudinal study

**DOI:** 10.1007/s00420-024-02108-5

**Published:** 2024-11-15

**Authors:** Christoph Golz, Miriam Hägerbäumer, Maisa Gerlach, André Meichtry, Gablu Kilcher, Karin Anne Peter, Eva Blozik

**Affiliations:** 1 School of Health Professions, Bern University of Applied Sciences, Murtenstrasse 10, Bern, 3008 Switzerland; 2 Department of Psychology, EURO-FH University of Applied Sciences, Hamburg, Germany; 3 Department Health Services Research, SWICA Healthcare Organization, Winterthur, Switzerland; 4 Careum School of Health, Kalaidos University of Applied Sciences, Zürich, Switzerland; 5 Institute of Primary Care, University and University Hospital Zürich, Zürich, Switzerland

**Keywords:** Presenteeism, Theory of planned behaviour, Attitudes, Vignettes, Occupational health, Longitudinal

## Abstract

**Purpose:**

This study uses the Theory of Planned Behaviour (TPB) to explore presenteeism, where individuals work despite being ill. The research seeks to understand how attitudes, subjective norms, and perceived behavioural control are associated with presenteeism behaviours.

**Methods:**

A longitudinal design was employed, involving 2814 employees from 16 companies. Data were collected using a survey on two measurement points, which included validated scales and vignettes to assess attitudes toward presenteeism. The data were analysed using cluster analysis and a linear mixed effects regression to evaluate the TPB model.

**Results:**

Three clusters of attitudes toward presenteeism were identified through cluster analysis. The model explained 27.8% of the variance in the fixed effects and 52.6% in the combined fixed and random effects. The regression model found associations between presenteeism and factors such as quantitative demands, work-privacy conflict, and leadership culture. Attitudes towards presenteeism were a strong predictor, with specific clusters showing differing propensities to work while ill.

**Conclusion:**

The study confirms the suitability of the TPB in explaining presenteeism. It highlights the importance of individual attitudes, subjective norms, and perceived behavioural control in shaping presenteeism. The findings suggest that promoting a health-supportive workplace culture, including open communication about illness, might reduce presenteeism.

**Supplementary Information:**

The online version contains supplementary material available at 10.1007/s00420-024-02108-5.

## Introduction

Presenteeism, defined as a behaviour in which individuals work despite feeling ill (Ruhle et al. 2019), can negatively impact employee well-being and organisational productivity (Johns 2010; Kigozi et al. 2017). It is a relevant factor for employees’ physical and mental health (Johns 2010) and has been shown to account for 52% of health-related production losses (Kigozi et al. 2017).

The decision to work despite feeling ill is influenced by the affected individuals, their type of work, and organisational factors (Lohaus and Habermann 2019). Remote work further complicates presenteeism behaviours (Miraglia and Johns 2016) since employees who feel ill may decide to stay at home but still work (Steidelmüller et al. 2020). Common reasons for continuing to work when ill include feelings of being indispensable, workload pressure, and not wanting to burden colleagues (Marklund et al. 2021). Although research has addressed antecedents and consequences of presenteeism and has provided comprehensive models (Johns 2010; Lohaus and Habermann 2019; Miraglia and Johns 2016), it has not sufficiently investigated the individual decision-making process that leads to presenteeism. This gap has been acknowledged by Lohaus and Habermann (2021), who focused on the role of individuals’ motivation in the decision-making process on being present or absent: Employees have been shown to base a conscious decision to go to work or not on their expected job performance, their expected reward and the perceived value associated with the reward. To study decision-making patterns, the authors developed and applied vignettes, defined as “a short, carefully constructed description of a person, object, or situation, representing a systematic combination of characteristics” (Atzmüller and Steiner 2010, p.128).

However, the nature of the expectancy theory underlying Lohaus and Haberman’s work also implies that individuals make a conscious decision to go to work or not after they determine for themselves that they are ill, a condition which is perceived and delineated differently by different individuals (Karanika-Murray and Cooper 2018). Expectancy theory, initially developed by Vroom (1964), suggests that people choose certain behaviours based on the expected outcomes, linking motivation and decision-making to the perceived value of rewards. Research suggests that illness perceptions vary between individuals, who are present or absent in case of illness. Employees affected by numerous and severe symptoms rather decide not to work (Fiorini 2024; Fiorini et al. 2020). In this context, research has aimed to identify health problems attributed to presenteeism or absenteeism. For example, gastritis was associated with the decision to go to work, but emotional problems predominantly led to absenteeism (Gosselin et al. 2013). However, “it is possible to have a disease without being ill” (Amzat and Razum 2014, p.29), and therefore, an approach associating diseases with presenteeism behaviour does not fully capture the underlying health behaviours.

In particular, the complex interplay between individual health perceptions and workplace behaviours has not been sufficiently integrated into the discussion. The Theory of Planned Behaviour (TPB), developed by Ajzen (1991) has been proposed as a suitable theoretical background to explain the interplay (Yusoff et al. 2021) and has been applied, e.g. in the context of presenteeism research with regard to individual attitudes towards COVID-19 prevention guidelines (Probst et al. 2021).

The TPB is a widely used psychological model for understanding and predicting human behaviour in specific contexts. The model posits that an individual’s behavioural intentions, which are the strongest predictors of actual behaviour, are influenced by three key components: attitude, subjective norms, and perceived behavioural control (Ajzen 1991).

The first component, attitude, refers to the individual’s positive or negative evaluation of performing a particular behaviour. For example, in the context of presenteeism, an employee might evaluate the behaviour of working while ill based on the perceived advantages (e.g., not falling behind on tasks) or disadvantages (e.g., worsening their health).

The second component, subjective norms, reflects the personal perception of the social expectations to adopt a given behaviour. This reflects how employees perceive their colleagues, supervisors, or workplace culture regarding working while ill. For example, subjective norms may be an employee’s perception that colleagues view absence for health reasons as a sign of weakness and lack of reliability or consider an affected person as lazy or unproductive (Yusoff et al. 2021), which can be summarized as the team or leadership culture.

The third component, perceived behavioural control, refers to personal beliefs as to how easy or difficult performing the behaviour is likely to be, which reflects the extent to which the behaviour is perceived to be under volitional control (Jimmieson et al. 2008). In their systematic review, Yusoff et al. (2021) also summarised different factors associated with the component perceived behavioural control, which were found to be job insecurity or quantitative demandsan.

In summary, findings on subjective social norms and perceived behavioural control and their relationship with presenteeism exist, but these factors have not yet been analysed on the theoretical basis of the TPB and with the inclusion of attitude. Therefore, the aim of this study was to measure individuals’ attitudes towards presenteeism and test the suitability of the TPB for explaining presenteeism behaviour. This comprised (1) a cluster analysis for the vignettes of attitude and (2) a linear mixed effects regression for evaluating the TPB model.

## Method

### Design

This study has a longitudinal design, with data collected at two time points (T1 and T2), separated by one year between March and April 2022 and 2023). We adhered to the STROBE reporting guideline for longitudinal studies (Von Elm et al. 2007). The checklist is included in supplementary file A. The reason for collecting data at two distinct time points, separated by one year, was to align with the recall interval of the Hägerbäumer presenteeism scale, which assesses presenteeism behaviour over the preceding 12 months (Hägerbäumer 2017). By spacing the surveys a year apart, we avoided any overlap in the reporting period, thus ensuring that each data point represented a distinct, non-overlapping 12-month timeframe. The longitudinal design is essential for examining the stability of presenteeism behaviour and identifying predictors that may change over time.

### Recruitment

A convenience sampling was conducted from 16 companies in the German-speaking part of Switzerland. The companies were identified from lists of working sectors published by national associations, such as the construction industry, healthcare, or education. Chief Executive Officers or the head of Human Resources received information about the project by email or telephone. The email included a flyer and a short film about the project. The participating companies exhibited variation in terms of size, with the following categorization: small (10–49 employees; *n* = 5), medium (50–249 employees; *n* = 6), and large (over 250 employees; *n* = 5). A total of *N* = 4’386 employees were eligible for participation (Gerlach et al. 2024).

### Study sample and data collection

For data collection, a single individual was appointed by each participating company to oversee the administration of the survey. This contact person distributed the questionnaires to all employees within the company. The first survey (T1) was distributed in spring 2022 and the second survey (T2) one year later. The questionnaire was available in German and English and could be completed online via Unipark^®^. Participants were given one month to complete the questionnaire, and a reminder was sent after two weeks. Each participant was assigned a unique code based on the first three letters of their mother’s and father’s names and birth months. This code allowed for the identification of unique cases.

### Questionnaire

The survey included questions on individual characteristics (age, country, and profession), the Hägerbäumer presenteeism scale (α = 0.89) (Hägerbäumer 2017), The dimension Supervision Distrust from the Presenteeism Climate Questionnaire (α = 0.89) (Ferreira et al. 2015), the Team Health Climate Questionnaire (α = 0.71) (Schulz et al. 2017), the scales quantitative/emotional demands, job insecurity, hiding emotions and work-privacy conflict from the Copenhagen Psychosocial Questionnaire III (COPSOQ) (Lincke et al. 2021) and self-developed vignettes to measure attitudes towards presenteeism.

**Fig. 1:**
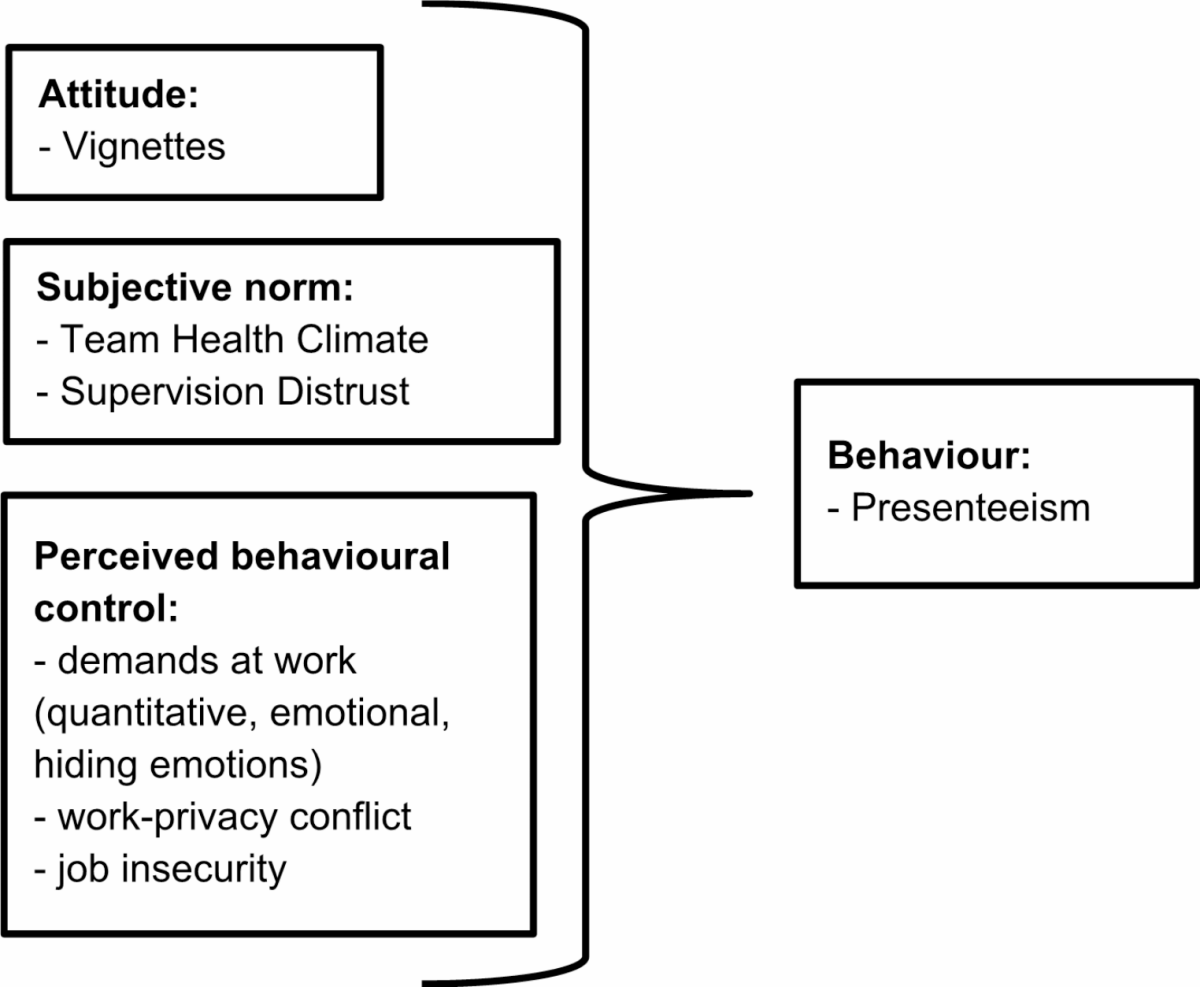
TPB model for presenteeism behaviour

These variables were selected based on their theoretical alignment with the components of the TPB model (Fig. 1). Specifically, attitudes toward presenteeism were captured through the vignettes, allowing us to assess individual evaluations of presenteeism behaviour in specific scenarios (Ajzen 1991). Subjective norms were measured using the Team Health Climate questionnaires and Supervision Distrust dimension of the Presenteeism Climate Questionnaire, reflecting the influence of social and organizational expectations on presenteeism (Yusoff et al. 2021). The Team Health Climate Questionnaire assesses the collective perception among team members about how their team handles health-related topics. Specifically, it measures the extent to which team members are concerned about health, care for one another’s well-being, and communicate about health issues within the team (Schulz et al. 2017). The Supervision Distrust measures the leadership culture with regard to the level of distrust employees feel from supervisors regarding their reasons for being absent. When employees perceive that their absence will be viewed with suspicion, they may choose presenteeism to avoid negative consequences (Ferreira et al. 2015).

Perceived behavioural control was measured through items assessing job insecurity, quantitative and emotional demands, and work-privacy conflict, which influence the perceived ease or difficulty of attending work while ill (Yusoff et al. 2021). We chose the COPSOQ III for measuring perceived behavioural control in this study because it is an internationally well-known and established tool for assessing psychosocial factors at work. The chosen dimensions comprise questions focussing on work-related factors, which may be associated the perceived volitional control. For example, known reasons for presenteeism behaviour are the workload or the fear of professional disadvantages in case of absence (Gerlach et al. 2024), which are asked with the COPSOQ questions, e.g. “Do you get behind with your work?” or “Are you worried about becoming unemployed?”.

The Hägerbäumer presenteeism scale comprises six items based on a five-point Likert scale, which 1 = “Never in case of illness”, 2 = “Rarely in case of illness”, 3 = “Sometimes in case of illness”, 4 = “Often in case of illness”, and 5 = “Very often in case of illness. In addition, it is possible to indicate that no illness occurred during the reference period (0 = ‘I was not ill’) (Hägerbäumer 2017). The Presenteeism Climate Questionnaire ranges between 0= “completely disagree” − 7 = “totally agree” with a high value indicating problematic leadership culture in dealing with presenteeism (Ferreira et al. 2015). The Team Health Climate Questionnaire (α = 0.71) ranges between 1= “disagree” − 4= “agree” with a higher value for positive handling in the team regarding presenteeism (Schulz et al. 2017). All item responses of the COPSOQ III scales were scored on a five-point Likert scale ranging from always - never/hardly never or to a very large extent - to a very small extent, with a high score indicating high demands. For interpretation of the scales, a mean score is calculated per case.

The vignettes were developed to describe situations in which an individual experiences common symptoms and consciously decides for or against working. We, therefore, developed vignettes to match common symptoms of the most prevalent diseases, such as cold or flu (Eccles 2023) or work-related, such as musculoskeletal disorders (Govaerts et al. 2021). The vignettes were developed by the research team and validated for understandability within a pretest of 10 employees working in companies from the German-speaking part of Switzerland. The vignettes could be rated on a scale ranging from 0 (very unlikely) to 100 (very likely):

1. Imagine you have a severe headache. How likely is it that you will still go to work?
2. If you wake up in the morning with a temperature of 38.5 °C and no other symptoms, how likely is it that you will still go to work?
3. If you wake up in the morning with a temperature of 38.5 °C and have a cough, a cold and an aching limb, how likely is it that you will still go to work?
4. If you wake up in the morning with severe stomach pain and no other symptoms, how likely would you be to go to work?
5. If you wake up in the morning with severe neck or back pain and no other symptoms, how likely would you be to go to work?

### Analysis

The COPSOQ III scales were transformed to a value range from 0 (minimum value) to 100 points (maximum value). No average score was calculated if less than half of the questions in a scale had been answered (Kristensen 2000).

First, descriptive statistics such as mean and standard deviation were calculated. Second, we performed agglomerative nesting cluster analysis to identify natural groupings within the data of the vignettes using data from the first survey (T1). We computed the dissimilarity matrix with euclidian metric considering the five vignettes. We then applied hierarchical clustering using three methods: average, complete linkage and Ward’s method. The resulting dendrograms were plotted to visualize the hierarchical structure of the clusters. To finalize the clustering process, we assigned cluster labels to each observation based on the chosen cluster solution. We used Principal Component Analysis (PCA) to visualize the clustered observations in a two- and three-component space, respectively, spanned by the first two (three) components of the PCA solution. Additionally, we calculated the Jaccard Index to assess the stability of the clustering solutions with bootstrap *n* = 1000. The value ranges between 0 and 1, where a value below 0.5 refers to low stability and a value of 1 to high stability. Values between 0.5 and 0.75 are moderate, and between 0.75 and 0.9 are good (Hennig 2008).

Third, we computed a linear mixed effects model with presenteeism as a dependent variable, the surveys (T1, T2) as a time effect, and the independent variables were selected theoretically based on the TPB model (Fig. 1). To address missing data, we used the “mice” package (Van Buuren and Groothuis-Oudshoorn 2011) for multivariate imputation by chained equations. We generated 5 imputed data sets to account for the uncertainty associated with missing data. The results were pooled, accounting for both within-imputation and between-imputation variability. Participants who responded that they had not been ill in the last 12 months were excluded from further analysis since they would not classify as behavioural presenteeism based on the underlying definition (Hägerbäumer 2017). A random intercept for subject was included to account for paired data.

Residual analysis was performed to assess model assumptions, that is, zero expectation, linearity, homoscedasticity and multicollinearity. In the case of evidence of heteroscedasticity, we repeated the pooling procedure by fitting a robust linear mixed effects model (Zeileis et al. 2019). For the quantitative analysis, we used R Version 4.4.1 (R Core Team 2024) and the packages “psych” (Revelle 2017), “cluster” (Maechler 2019) and “lme4” (Bates et al. 2015).

## Results

The study sample consisted of 2814 employees from 16 companies. Overall, 64% (*n* = 1799) reported being ill at least once in the last 12 months. Most participants worked in the insurance sector (T1 = 65.6%; T2 = 79.9%). Most participants were female (T1 = 64.2%; T2 = 63.4%) with a mean age of 49.3 years (SD = 11.8); they had an average of 13.15 (SD = 10.4) years of professional experience and 8.8 (SD = 7.5) years working in their current company. Most participants originated from Switzerland (T1 = 83.9%; T2 = 83.8%) (see Table 1). For the first survey (T1), the response rate was 47.2%, and in the second survey (T2) 22.7%.

**Table 1:** Sample characteristics

	T1 (*N* = 2071)		T2 (*N* = 743)	
Response rate				
Characteristics	Mean (SD)	N (%)	Mean (SD)	N (%)
Age	49.4 (12.1)		49.2 (11.5)	
Sex				
Female		1294 (62.5)		471 (63.4)
Male		703 (34.0)		248 (33.4)
Missing		74 (3.6)		24 (3.2)
Education				
No education		18 (0.9)		5 (0.7)
Secondary II		844 (40.8)		365 (49.1)
Tertiary B		410 (19.8)		176 (23.7)
BSc		210 (10.1)		101 (13.6)
MSc		147 (7.1)		63 (8.5)
PhD		46 (2.2)		30 (4.0)
Missing		396 (19.1)		3 (0.4)
Professional experience	12.9 (10.6)		13.4 (10.2)	
Current position (years)	8.2 (7.5)		9.5 (7.5)	
Sector				
Insurance		1340 (64.7)		594 (79.9)
Healthcare		249 (12.0)		0 (0.0)
Education		103 (5.0)		34 (4.6)
Informatics		141 (6.8)		51 (6.9)
Social Services		128 (6.2)		50 (6.7)
Manufacture		88 (4.2)		14 (1.9)
Production of printed products		18 (0.9)		0 (0.0)
Gastronomy		4 (0.2)		0 (0.0)
Origin				
Switzerland		1738 (83.9)		623 (83.8)
Other countries		333 (16.1)		120 (16.2)

### Cluster analysis

**Fig. 2:**
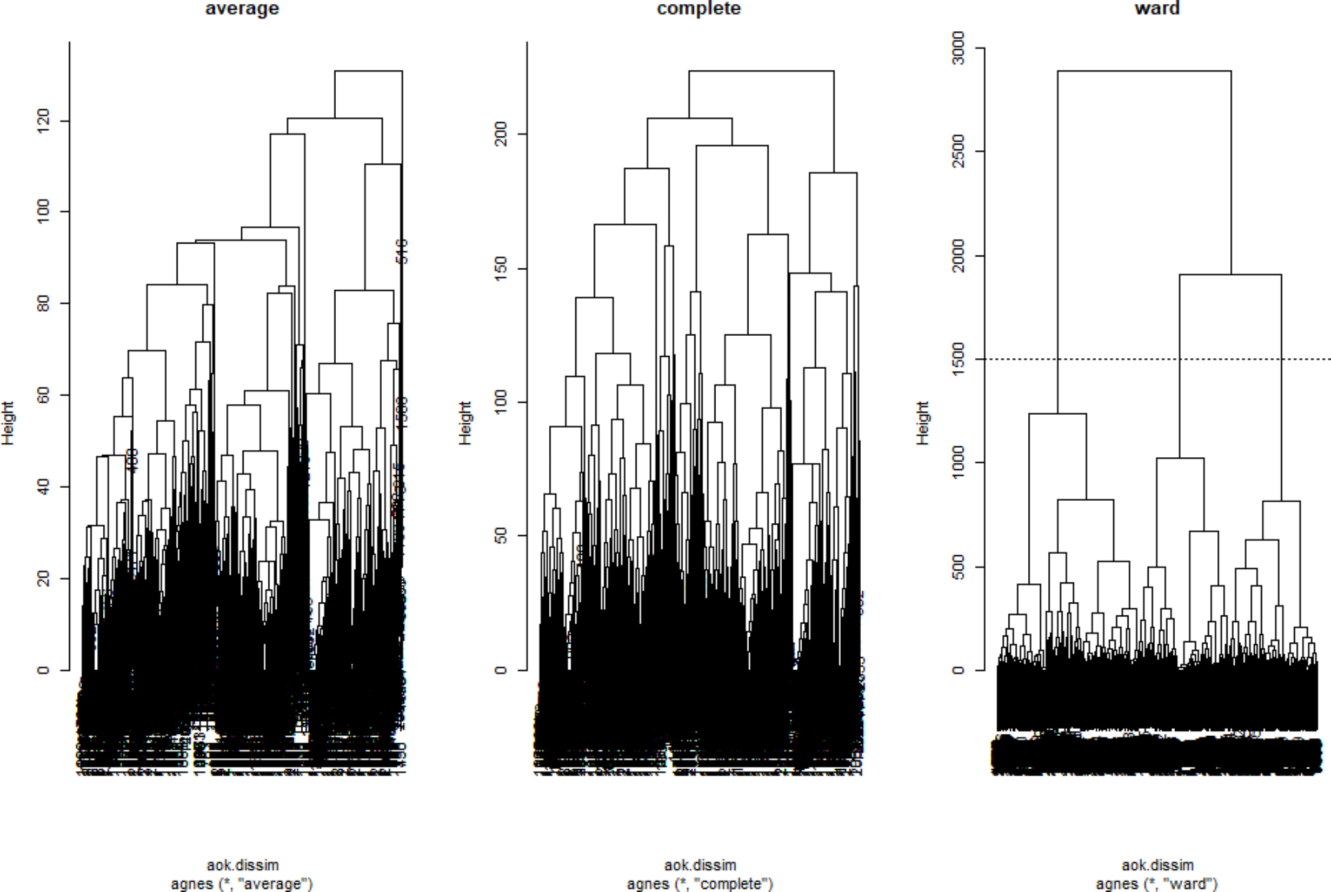
Dendograms of the three applied methods average, complete and ward

The cluster analysis was conducted with 2’071 complete cases. Figure 2 shows the final dendograms of the individual variables (Fig. 2). Among the three computed plots, Ward’s method resulted in the best interpretable output.

It minimised the total within-cluster variance and indicated a more distinct separation of clusters, suggesting that this method was more suitable for our data. We decided to partition the data into three clusters (A, B, C), as the dendrogram ‘ward’ indicated. The figure is to be understood as follows: The participants are compared with each other in terms of their assessment of all five items and, if they have the same, are merged. This results in a grouping with three clear groups at the stem. Across all participants, three groups are similar in their assessment of the five items. Group A comprises *n* = 912 employees, followed by Group B with *n* = 580 and Group C with *n* = 579.

In Table 2, the descriptives of the vignettes per cluster are summarised. Employees assigned to cluster A have higher mean values across all vignettes than those assigned to the two other clusters. In three of the five vignettes, employees assigned to cluster B have the lowest mean values (1, 4 and 5). Employees assigned to cluster C have high mean values in three of the five vignettes and the lowest in two (vignettes 2 and 3). Vignette three has the lowest mean value across all clusters (Table 2).

**Table 2:** Descriptives of vignettes per cluster (Mean, SD)

Cluster	Vignette 1	Vignette 2	Vignette 3	Vignette 4	Vignette 5
A	80.7 (23.2)	72.5 (22.3)	37.4 (30.9)	75.7 (23.7)	87.7 (16.7)
B	32.6 (33.3)	14.4 (21.3)	3.04 (7.87)	12.4 (15.2)	35.2 (27.6)
C	70.0 (26.9)	7.21 (10.1)	1.63 (5.04)	62.1 (28.2)	83.7 (17.1)

**Fig. 3:**
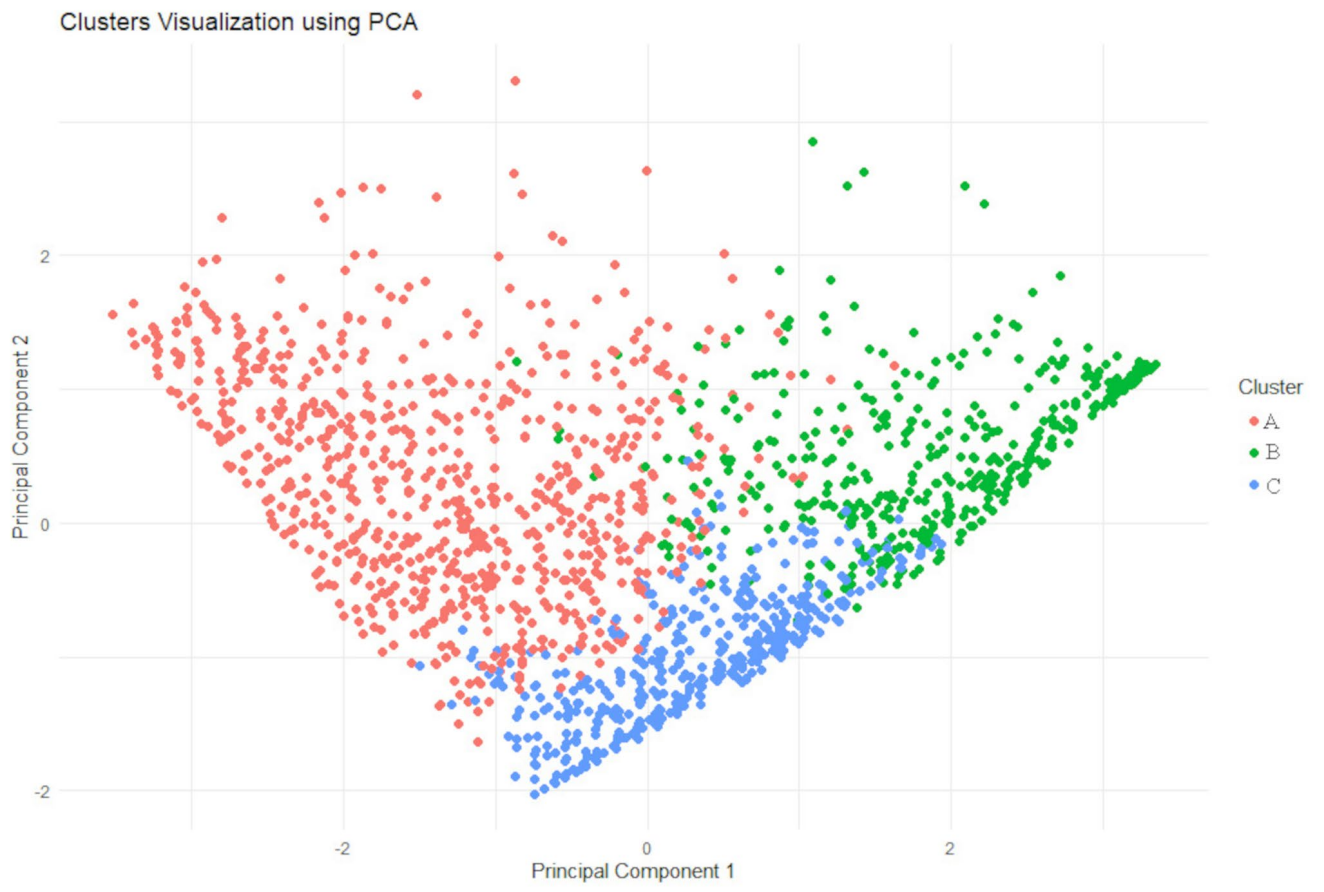
Clustered Observations in the plane spanned by the first two principal components

**Fig. 4:**
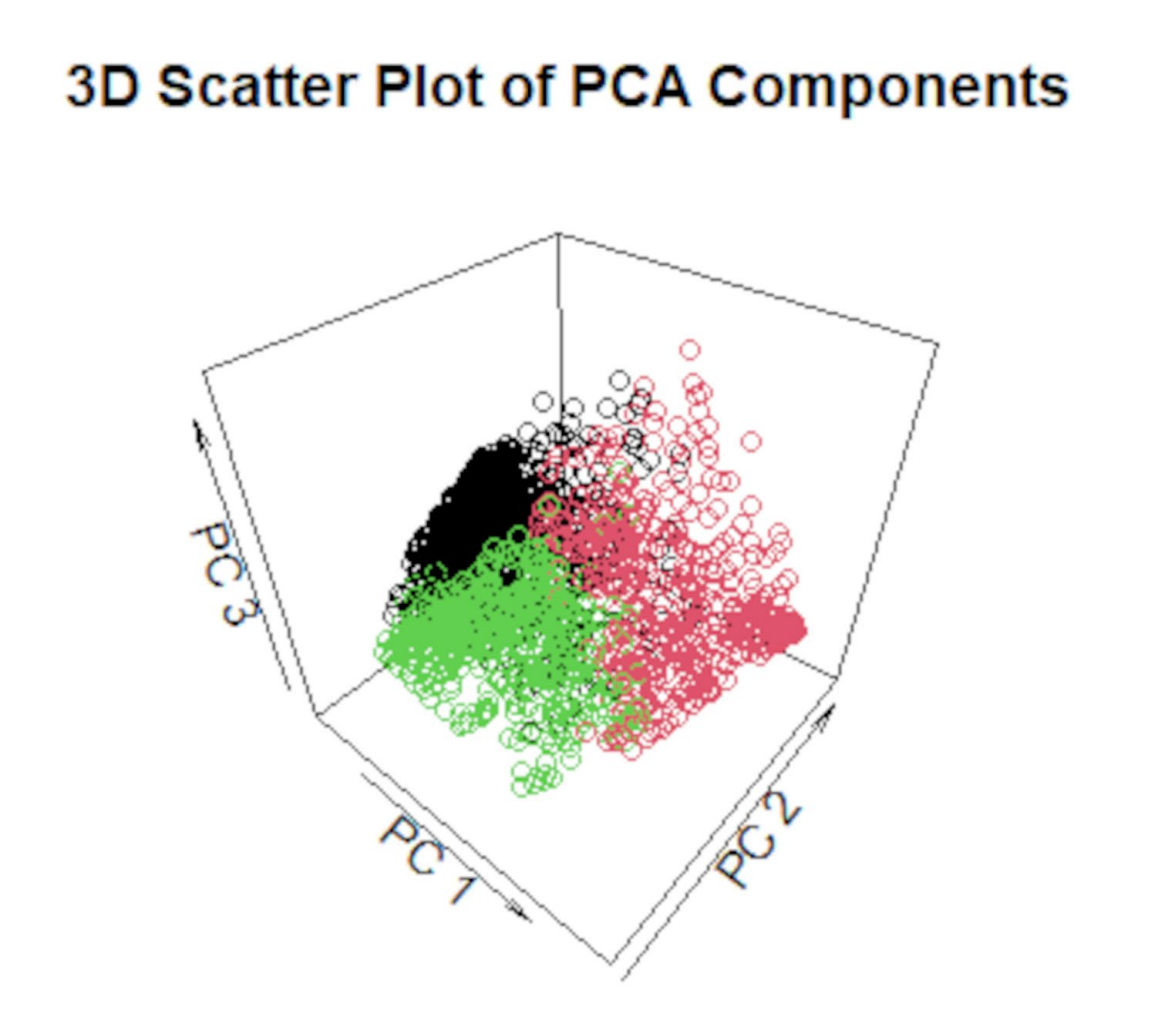
Clustered Observations in the space spanned by the first three principal components

Figure 3 shows the clustered observations in the plane spanned by the first two components of the PCA solution distinguished by color, and Fig. 4 the clustered observations in a three-component space. By plotting the clusters in the space of the first two principal components, we visualized how the observations naturally group together based on their similarities. This 2D representation provides an intuitive way to observe the relative positions and separations of the clusters, highlighting key patterns and trends in the data. The distinct separation (or overlap) of clusters in this figure offers insights into how well-defined the clusters are, with clear separation indicating robust clustering.

Figure 4 shows a clear separation between clusters, which suggests the robustness of the clustering method applied. Adding the third component provided information on additional variance that may not have been visible in the 2D plane. This 3D visualization enhances the understanding of cluster separations and relationships between observations, offering more depth to the analysis. The first principal component (PC1) explains 59.0% of the total variance, the PC2 explains an additional 18.8%, and the PC3 provides an additional explanation of the total variance with 9.7%, resulting in an explanation of total variance with three components of 87.5%. The Jaccard Index is satisfactory for all three clusters, with A = 0.6, B = 0.72, and C = 0.6.

In summary, the distinction between the clusters is clear. Cluster A represents a group that is more likely to engage in presenteeism, while Cluster B is the opposite, and Cluster C is more conditional based on symptom severity.

### Regression analysis

The analysis showed heteroscedasticity and therefore we computed a robust linear mixed effects model. Table 3 summarises the results of the robust linear mixed effects regression model for presenteeism. Residual analysis showed no evidence of violation of the other model assumptions. The model explained 27.8% of the variance in the fixed effects (Marginal R² = 0.278) and 52.6% of the variance in the fixed and random effects combined (Conditional R² = 0.526). The random intercept for unique subjects accounted for a substantial portion of the variance (τ00 id = 0.22), with an Intraclass Correlation Coefficient (ICC) of 0.34, indicating that 34% of the total variance is attributable to individual differences.

Among the factors for perceived behavioural control, job insecurity (B = 0.003, *p* = < 0.001), quantitative demands (B = 0.004, *p* <.001) and work-privacy conflict (B = 0.007, *p* <.001) were significant. For social norm, a positive leadership culture (B = 0.14, *p* <.001) and a positive team health culture (B = -0.07., *p* =.05) were significant. Regarding the attitude, being assigned to cluster B (B = -0.69, *p* <.001) or cluster C (B = -0.53, *p* <.001) was found to be negatively associated with presenteeism compared to employees assigned to cluster A. The values are to be understood as follows: With an increase of e.g. 1 point in Work-Privacy Conflict (range 0–100), Presenteeism increases by 0.007 (range 1–5). After controlling for other covariates in the model, the dependent variable did not significantly change between the two measurement points. Therefore, the levels of presenteeism remained stable over the period of observation.

**Table 3:** Pooled estimates of robust linear models with presenteeism as the outcome

Coefficient	B	Std. Error	*p*-value	CI (95%)	
Intercept	1.94	0.100	< 0.001	1.73–2.15	
Job Insecurity	0.003	0.000	< 0.001	0.002–0.005	
Quantitative Demands	0.004	0.000	< 0.001	0.002–0.007	
Emotional Demands	-0.000	0.000	0.57	-0.002–0.001	
Hiding Emotions	0.001	0.000	0.14	-0.000–0.003	
Work-Privacy Conflict	0.007	0.001	< 0.001	0.005–0.01	
Team Health Culture	-0.07	0.024	0.006	-0.12– -0.02	
Leadership Culture	0.14	0.02	< 0.001	0.10–0.17	
Cluster B	-0.69	0.04	< 0.001	-0.78– -0.60	
Cluster C	-0.53	0.04	< 0.001	-0.62– -0.45	
Time	-0.01	0.04	0.72	-0.09–0.06	
Random Effects					
σ2	0.42				
τ00 subject	0.22				
ICC	0.34				
N subject	2310				

Hägerbäumer Presenteeism scale ranges between 1 (never in case of illness) and 5 (very often in case of illness)

Reference category for cluster: cluster A

## Discussion

This study aimed to measure individuals’ attitudes towards presenteeism and test the suitability of the TPB for explaining presenteeism behaviour. The cluster analysis of the developed vignettes to measure the individuals’ attitude toward presenteeism resulted in a satisfactory three-component solution. The three components can be clearly distinguished, and the validation by applying PCA and the Jaccard Index resulted in a good fit.

The three clusters show clear differences regarding presenteeism behaviour. Individuals assigned to cluster A show a rather positive attitude towards presenteeism behaviour across all vignettes compared to the other clusters. Individuals assigned to cluster B show a rather negative attitude towards presenteeism behaviour and decide to call in sick when they perceive common symptoms. Individuals assigned to cluster C somehow decide to call in sick when they have an objective indicator, such as a temperature of 38.5 °C (vignettes 2–3), and without such objective indicator, a positive attitude towards presenteeism behaviour.

This pattern could be explained by the different lay concepts of health by Faltermaier (1994). He described three forms of health behaviour, which could be assigned to the above-described clusters. Cluster A is characterised by action and performance-oriented health awareness. Individuals in this health concept ignore health complaints until their ability to work is impaired. The importance of health and health maintenance is low, and work is a priority (Faltermaier 2023). Cluster B is described by the health concept of psychologically influenced and multidimensional health awareness. Individual health is a high priority among individuals, and they strive for well-being, peace, balance, and the absence of pain and discomfort. The individuals have high health awareness, and symptoms are understood as signals of overload and psychological conflicts (Faltermaier 2023). Cluster C may be characterised by the health concept organic-medical health awareness. Health is understood as the absence of illness and illness is perceived if identifiable symptoms are present. The importance of health is also low in this group (Faltermaier 2023).

However, this comparison is purely hypothetical and requires further detailed analysis to determine whether the theoretical framing of the identified cluster is correct. Thus, it would be necessary to identify the characteristics of the three groups. For example, the theory assumes that women, in particular, have a higher level of health awareness and are, therefore, more likely to be assigned to the health concept of psychologically influenced and multidimensional health awareness (Faltermaier 1994). In contrast, the literature shows a rather heterogeneous picture of presenteeism behaviour differences between females and males (Webster et al. 2019). Regarding presenteeism, the profession and the setting seem more decisive, such as social professions (Aronsson et al. 2000; Chambers et al. 2017), which naturally have a disproportionate number of women.

We were able to evaluate the suitability of the TPB on presenteeism behaviour. As the theory explains, attitude, subjective norm, and perceived behavioural control are associated with the behaviour. Our model explains 29.6% of the variance in the fixed effects and 51.6% of the variance in the fixed and random effects combined and the higher value compared to the marginal R² suggests that a significant portion of the variability in presenteeism is also due to individual differences captured by the random effects (subjects).

The individual’s attitude seems to be a highly relevant factor for presenteeism behaviour. As discussed in the paragraph above, the individuals’ health awareness may form the attitude. This awareness is mutually shaped by lifestyle. However, it is not static and can change due to dealing with health issues (Faltermaier 2023).

Subjective norms, such as workplace expectations or peer behaviours, significantly influence whether employees attend work while ill. This insight underlines the importance of communicating about illness in the workplace. A health culture in the company that promotes a discussion about one’s health appears to be a potentially effective basis for reducing presenteeism.

Management plays a key role in the formation of the presenteeism climate. They have, in addition, a role model, and research showed that their presenteeism behaviour influences the employees’ behaviour (Dietz et al. 2020). The results of the mixed effects regression show that management’s handling of illness is linked to presenteeism. The feeling of being able to talk to direct superiors about illness was found to be associated with lower presenteeism. This finding aligns with other research on leadership and presenteeism behaviour (Ferreira et al. 2015), particularly leadership styles such as transformational (Cicek and Kilinc 2020), inclusive (Qian and Wang 2023) or health-oriented (Vonderlin et al. 2021). Inclusive leadership is characterised by openness and aims to build psychological safety (Cicek and Kilinc 2020); it may provide a fruitful ground for establishing a positive health culture at work. However, there also appears to be a risk of increased presenteeism behaviour due to such management styles under certain conditions, such as increased subjective obligation (Qian and Wang 2023).

Perceived behavioural control, reflecting factors like job insecurity and workload, also played a key role, affecting employees’ perceived ability to make autonomous decisions regarding their health. Higher perceived job insecurity and quantitative demands were associated with a higher presenteeism behaviour.

The findings underscore the importance of addressing both social and organizational expectations (subjective norms) and workplace demands (perceived behavioural control) in efforts to mitigate presenteeism. By focusing on both aspects, organizations can create environments that support healthier decision-making, allowing employees to stay home when ill without fearing negative consequences.

Measures are therefore needed to enable leaders to communicate with their employees about the subjectiveness of illness. They must be aware of the adverse consequences that proposed leadership styles can have. In this context, research is needed to determine how open communication and a health-promoting culture at work can be implemented without achieving the opposite results in presenteeism through increased commitment and subjective obligation. One effective approach to improving workplace culture and addressing presenteeism is implementing health-oriented leadership interventions. Health-oriented leadership emphasizes the role of supervisors in promoting health by focusing on self-care and staff care. A specific example is mindfulness- and skill-based health-oriented leadership intervention developed by Vonderlin et al. (2021), which demonstrated significant improvements in supervisors’ mental health and health-promoting behaviours. Regarding a healthy work culture, research suggests that improving working conditions is related to improved presenteeism. A recent systematic review concluded that only multicomponent programs on different organizational levels positively affected presenteeism (Støren and Grønningsæter 2024). Such successful multi-component programs comprised individual health and lifestyle. In addition, applying participatory processes for the program implementation was identified as favourable, which in turn is associated with the leader’s behaviour.

### Strengths and limitations

This article follows the STROBE reporting guidelines for longitudinal studies (Von Elm et al. 2007). The study also benefits from a large sample size, facilitating robust statistical analyses and providing deeper insights into our research questions. This extensive sample size improved our ability to accurately identify associations within the longitudinal data and reduced the likelihood of random results, thereby enhancing the study’s internal validity. Additionally, the study’s strength lies in its use of valid and reliable scales. The longitudinal design offers distinct advantages in understanding presenteeism behaviour over time. Unlike cross-sectional studies, which provide a snapshot of behaviour at a single point, our longitudinal approach allows us to observe changes and trends in presenteeism behaviour, providing deeper insights into how attitudes, subjective norms, and perceived behavioural control evolve and influence presenteeism over time.

One limitation of the study is that the complete TPB could not be applied, as no intention was measured, but rather presenteeism behaviour directly. We can, therefore, only make a statement about the extent to which the individual factors are directly related to the behaviour, but not which intention they generate, which ultimately leads to the behaviour. While interaction effects, such as those between subjective norm and perceived behavioural control, may offer additional insights into the dynamics of presenteeism, these were not included in the present analysis. Our primary aim was to evaluate the direct contributions of attitude, subjective norm and perceived behavioural control on presenteeism. Future research could explore these interaction effects further to refine the understanding of the model in this context.

The vignettes developed also only describe physical symptoms of common illnesses. Psychological impairments, such as depressive or burnout symptoms, are not currently included. However, mental health is found to be equally relevant in the context of presenteeism behaviour (Ruhle et al. 2019). Future research should expand the vignettes to include this perspective, particularly about common psychological impairments among the working population, such as burnout symptoms (Van Der Molen et al. 2020). The addition may contribute to an improvement of the identified clusters.

Furthermore, the voluntary nature of the questionnaire may have led to the underrepresentation of specific employee groups. This could result in biased outcomes, as the perspectives of these underrepresented groups might differ significantly from those who chose to participate. Consequently, the findings may not fully capture the diversity of experiences and opinions within the entire workforce, potentially limiting the generalizability of the results. However, the differences identified in the cluster analysis indicate that, despite the survey method, a sufficiently broad range of participants with different attitudes towards presenteeism could be included. Additionally, the response rate decreased from 40 to 20% between the measurement points due to companies who decided not to participate in a second round. Reasons for dropouts of the companies were too many inquiries, such as from research and annual job satisfaction surveys and the appointment of a new manager, resulting in a shift in priorities. It is also important to note that the sampling was drawn primarily from specific industries with different proportions, which may limit the generalizability of the findings across other sectors. The workplace culture, job demands, and presenteeism patterns in other industries differ (Marklund et al. 2021), and future research could address this by including a broader range of sectors to enhance the applicability of the findings.

## Conclusions

In this study, we explored presenteeism using the TPB and showed that the theory is suitable for elaborating presenteeism. Although research has already addressed attitudes to presenteeism behaviour, research on presenteeism behaviour is still in its early stages. Research on presenteeism involving the TPB is thus marginal, and our contribution provides insights that may direct further research. We developed vignettes, which allow to allocate individuals in three distinct clusters with different attitudes toward presenteeism behaviour. We showed a strong association between attitudes and presenteeism as well as subjective norms and perceived behavioural control. By employing a longitudinal design, the study addresses the gaps in the existing literature, which often relies on cross-sectional data, thereby enhancing the validity and applicability of the findings. The innovative use of vignettes for clustering individuals by their presenteeism-related attitudes represents a novel methodological contribution that extends presenteeism research. This methodological strength adds a significant dimension to the understanding of presenteeism, providing a comprehensive view that can inform theoretical advancements and practical interventions in occupational health. Further research is needed to apply the vignettes in other samples and elaborate on the potential implications for suitable measures in reducing presenteeism.

## Electronic supplementary material

Below is the link to the electronic supplementary material.

Supplementary Material 1

## Data Availability

The raw dataset analyzed in the current study is available from the corresponding author upon reasonable request.

## References

[CR1] Ajzen I (1991) The theory of planned behavior. Organ Behav Hum Decis Process 50(2):179–211

[CR2] Amzat J, Razum O (2014) Health, Disease, and Illness as Conceptual Tools. In J. Amzat & O. Razum, Medical Sociology in Africa (S. 21–37). Springer International Publishing. 10.1007/978-3-319-03986-2_2

[CR3] Aronsson G, Gustafsson K, Dallner M (2000) Sick but yet at work. An empirical study of sickness presenteeism. J Epidemiol Community Health 54(7):502–509 10846192 10.1136/jech.54.7.502 PMC1731716

[CR4] Atzmüller C, Steiner PM (2010) Experimental vignette studies in survey research. Methodology

[CR5] Bates D, Maechler M, Bolker B, Walker S, Christensen RHB, Singmann H, Dai B, Grothendieck G, Green P, Bolker MB (2015) Package ‘lme4’. convergence, 12(1):2

[CR6] Chambers C, Frampton C, Barclay M (2017) Presenteeism in the New Zealand senior medical workforce-a mixed-methods analysis. NZ Med J 130(1449):10–21 28178725

[CR7] Cicek B, Kilinc E (2020) The mediating role of transformational leadership in the effect of technostress on presenteeism and intention to leave. Bus Econ Res J 11(2):555–570

[CR8] Dietz C, Zacher H, Scheel T, Otto K, Rigotti T (2020) Leaders as role models: effects of leader presenteeism on employee presenteeism and sick leave. Work Stress 34(3):300–322. 10.1080/02678373.2020.1728420

[CR9] Eccles R (2023) Common cold. Front Allergy 4:1224988. 10.3389/falgy.2023.1224988 37426629 10.3389/falgy.2023.1224988 PMC10324571

[CR10] Faltermaier T (1994) Gesundheitsbewußtsein Und Gesundheitshandeln. Beltz, Psychologie-Verl.-Union

[CR11] Faltermaier T (2023) Gesundheitspsychologie. Kohlhammer

[CR12] Ferreira AI, Martinez LF, Cooper C, Gui DM (2015) LMX as a negative predictor of presenteeism climate. J Organizational Effectiveness: People Perform 2(3):282–302. 10.1108/joepp-02-2015-0005

[CR13] Fiorini LA (2024) Remote workers’ reasons for changed levels of Absenteeism, Presenteeism and Working outside agreed hours during the COVID-19 pandemic. Sage Open 14(1):21582440241240636. 10.1177/21582440241240636

[CR14] Fiorini LA, Houdmont J, Griffiths A (2020) Nurses’ illness perceptions during presenteeism and absenteeism. Occup Med 70(2):101–106 10.1093/occmed/kqaa012 31961931

[CR15] Gerlach M, Blozik E, Meichtry A, Hägerbäumer M, Kilcher G, Golz C (2024) Factors of presenteeism and its association with detrimental effects among employees in Switzerland working in different sectors– a cross-sectional study using a multi-item instrument. Int Arch Occup Environ Health. 10.1007/s00420-024-02083-x 10.1007/s00420-024-02083-x PMC11416405 38951215

[CR16] Gosselin E, Lemyre L, Corneil W (2013) Presenteeism and absenteeism: differentiated understanding of related phenomena. J Occup Health Psychol 18(1):75–86. 10.1037/a0030932 23276197 10.1037/a0030932

[CR17] Govaerts R, Tassignon B, Ghillebert J, Serrien B, De Bock S, Ampe T, Makrini E, Vanderborght I, Meeusen B, R., De Pauw K (2021) Prevalence and incidence of work-related musculoskeletal disorders in secondary industries of 21st century Europe: a systematic review and meta-analysis. BMC Musculoskelet Disord 22:1–30 34465326 10.1186/s12891-021-04615-9 PMC8408961

[CR18] Hägerbäumer M (2017) Risikofaktor Präsentismus: Hintergründe Und Auswirkungen Des Arbeitens Trotz Krankheit. Springer

[CR19] Hennig C (2008) Dissolution point and isolation robustness: robustness criteria for general cluster analysis methods. J Multivar Anal 99(6):1154–1176. 10.1016/j.jmva.2007.07.002

[CR20] Jimmieson NL, Peach M, White KM (2008) Utilizing the theory of Planned Behavior to inform Change Management: An Investigation of Employee intentions to support organizational change. J Appl Behav Sci 44(2):237–262. 10.1177/0021886307312773

[CR21] Johns G (2010) Presenteeism in the workplace: a review and research agenda. J Organizational Behav 31(4):519–542. 10.1002/job.630

[CR22] Karanika-Murray M, Cooper CL (2018) Presenteeism: An introduction to a prevailing global phenomenon

[CR23] Kigozi J, Jowett S, Lewis M, Barton P, Coast J (2017) The estimation and inclusion of presenteeism costs in applied economic evaluation: a systematic review. Value Health 20(3):496–506. 10.1016/j.jval.2016.12.006 28292496 10.1016/j.jval.2016.12.006

[CR24] Kristensen TS (2000) A new tool for assessing psychosocial factors at work: the Copenhagen Psychosocial Questionnaire. National Institute of Health

[CR25] Lincke H-J, Vomstein M, Lindner A, Nolle I, Häberle N, Haug A, Nübling M (2021) COPSOQ III in Germany: validation of a standard instrument to measure psychosocial factors at work. J Occup Med Toxicol 16(1):1–15 34784940 10.1186/s12995-021-00331-1 PMC8594291

[CR26] Lohaus D, Habermann W (2019) Presenteeism: a review and research directions. Hum Resource Manage Rev 29(1):43–58. 10.1016/j.hrmr.2018.02.010

[CR27] Lohaus D, Habermann W (2021) Understanding the decision-making process between presenteeism and absenteeism. Front Psychol 12:716925 34354653 10.3389/fpsyg.2021.716925 PMC8329342

[CR28] Maechler M (2019) Finding groups in data: cluster analysis extended Rousseeuw. R Package Version 2(0):242–248

[CR29] Marklund S, Gustafsson K, Bergström G, Leineweber C (2021) Reasons for presenteeism in different occupational branches in Sweden: a population based cross–sectional study. Int Arch Occup Environ Health 94:1385–1395 33914162 10.1007/s00420-021-01701-2 PMC8292261

[CR30] Miraglia M, Johns G (2016) Going to work ill: a meta-analysis of the correlates of presenteeism and a dual-path model. J Occup Health Psychol 21(3):261–283. 10.1037/ocp0000015 26550958 10.1037/ocp0000015

[CR31] Probst TM, Lee HJ, Bazzoli A, Jenkins MR, Bettac EL (2021) Work and non-work sickness presenteeism: the role of workplace COVID-19 climate. J Occup Environ Med 63(8):713–718 33973931 10.1097/JOM.0000000000002240 PMC8327763

[CR32] Qian Z, Wang D (2023) The double-edged sword effect of inclusive leadership on employee presenteeism. Curr Psychol 42(27):23400–23412. 10.1007/s12144-022-03493-1

[CR33] R Core Team (2024) R: a Language and Environment for Statistical Computing. R Foundation for Statistical Computing. https://www.R-project.org/

[CR34] Revelle WR (2017) Psych: Procedures for personality and psychological research

[CR35] Ruhle SA, Breitsohl H, Aboagye E, Baba V, Biron C, Correia Leal C, Dietz C, Ferreira AI, Gerich J, Johns G, Karanika-Murray M, Lohaus D, Løkke A, Lopes SL, Martinez LF, Miraglia M, Muschalla B, Poethke U, Sarwat N, Yang T (2019) To work, or not to work, that is the question– recent trends and avenues for research on presenteeism. Eur J Work Organizational Psychol 29(3):344–363. 10.1080/1359432x.2019.1704734

[CR36] Schulz H, Zacher H, Lippke S (2017) The Importance of Team Health Climate for Health-Related Outcomes of White-Collar Workers. Front Psychol 8:74. 10.3389/fpsyg.2017.00074 28194126 10.3389/fpsyg.2017.00074 PMC5276847

[CR37] Steidelmüller C, Meyer S-C, Müller G (2020) Home-based telework and Presenteeism Across Europe. J Occup Environ Med 62(12):998–1005. 10.1097/JOM.0000000000001992 32796258 10.1097/JOM.0000000000001992 PMC7720871

[CR38] Støren PG, Grønningsæter H (2024) Do worksite health promotion programs (WHPP) influence presenteeism among employees? A systematic review1. Work 77(1):85–102. 10.3233/WOR-220115 37483043 10.3233/WOR-220115

[CR39] Van Buuren S, Groothuis-Oudshoorn K (2011) Mice: Multivariate imputation by chained equations in R. J Stat Softw 45:1–67

[CR40] Van Der Molen HF, Nieuwenhuijsen K, Frings-Dresen MHW, De Groene G (2020) Work-related psychosocial risk factors for stress-related mental disorders: an updated systematic review and meta-analysis. BMJ Open 10(7):e034849. 10.1136/bmjopen-2019-034849 10.1136/bmjopen-2019-034849 PMC7337889 32624469

[CR41] Von Elm E, Altman DG, Egger M, Pocock SJ, Gøtzsche PC, Vandenbroucke JP (2007) The strengthening the reporting of Observational studies in Epidemiology (STROBE) statement: guidelines for reporting observational studies. Lancet 370(9596):1453–1457. 10.1016/S0140-6736(07)61602-X 18064739 10.1016/S0140-6736(07)61602-X

[CR42] Vonderlin R, Müller G, Schmidt B, Biermann M, Kleindienst N, Bohus M, Lyssenko L (2021) Effectiveness of a mindfulness-and skill-based health-promoting leadership intervention on supervisor and employee levels: a quasi-experimental multisite field study. J Occup Health Psychol 10.1037/ocp0000301 34591521

[CR43] Vroom VH (1964) Work and motivation. John Willey & Sons

[CR44] Webster RK, Liu R, Karimullina K, Hall I, Amlot R, Rubin GJ (2019) A systematic review of infectious illness presenteeism: prevalence, reasons and risk factors. BMC Public Health 19(1):1–13 31226966 10.1186/s12889-019-7138-x PMC6588911

[CR45] Yusoff HM, Sobri HNM, Sundaram V (2021) Factors influencing intention to work while ill: a systematic review. Am J Health Behav 45(6):1016–1030 34969413 10.5993/AJHB.45.6.6

[CR46] Zeileis A, Lumley T, Berger S, Graham N, Zeileis MA (2019) Package ‘sandwich’. R Package Version, 2.5–1

